# Fatal and Nonfatal Injuries Involving Fishing Vessel Winches — Southern Shrimp Fleet, United States, 2000–2011

**Published:** 2013-03-08

**Authors:** Devin Lucas, Chelsea Woodward, Jennifer Lincoln

**Affiliations:** Alaska Pacific Office, National Institute for Occupational Safety and Health, CDC

Workers in the commercial fishing industry have the highest occupational fatality rate in the United States, nearly 35 times higher in 2011 than the rate for all U.S. workers (1). During 2000–2009, a total of 504 fishermen were killed in the U.S. fishing industry, most commonly by drowning as a result of vessels sinking (51%) and falls overboard (30%). Another 10% of fatalities (51 deaths) were caused by injuries sustained onboard vessels, such as entanglement in machinery (2). This type of fatality occurred most often in the Gulf of Mexico. To analyze fatal and nonfatal injuries involving deck winches in the Southern shrimp fleet during 2000–2011, CDC obtained data from its Commercial Fishing Incident Database and the U.S. Coast Guard. Injury patterns were examined, and risk ratios (RRs) were calculated to compare the probability of fatal outcomes from incidents involving different winch mechanisms and operating situations. During 2000–2011, eight fatal and 27 work-related injuries involving deck winches occurred in the Southern shrimp fleet, which operates in the Gulf of Mexico and off the Atlantic coast from Florida to North Carolina.* Injuries involving the winch drum had a higher risk for fatal outcomes compared with injuries involving the winch cathead. Fatal outcomes also were associated with being alone on the vessel and being alone on deck. Interventions to prevent deck winch injuries might include guarding of winch drums and catheads, avoiding working alone on deck, not wearing baggy clothing, and improvements to cable winding guides. Training of deckhands in first aid and emergency procedures might reduce the severity of injuries when entanglements occur.

Data on fatal injuries in the Southern shrimp fleet involving winches during 2000–2011 were identified in the Commercial Fishing Incident Database, a CDC surveillance system. Data on nonfatal injuries during the same period were collected from a U.S. Coast Guard database. The nature of injury and body part injured were coded using the Occupational Injury and Illness Classification System (3). Injury severity was coded with the Abbreviated Injury Scale (4). Investigators also conducted site visits to major shrimp fishing ports in Louisiana during October 2012, in partnership with local U.S. Coast Guard personnel. Winches on shrimp boats were observed in operation and examined to understand their mechanical features.

During 2000–2011, a total of 35 work-related injuries involving deck winches occurred in the Southern shrimp fleet, an average of three per year. Winch injuries occurred throughout the year, with no notable seasonal pattern. Among the 32 injuries for which time of occurrence was available, injuries occurred equally during daylight hours (16) and darkness (16). Twenty-six (74%) of the injury incidents occurred in the Gulf of Mexico, including 17 off the coasts of Texas and Louisiana; nine (26%) were scattered around the coasts of Florida. The median distance from shore of the vessels at the time of injury was 9 miles (range: 0.2–90.0 miles). Type of shrimp boat was known for 31 of the injuries; 28 (90%) of the injuries occurred on side trawlers, and three (10%) on skimmers. The median age of the vessels was 19 years (range: 5–32 years), and the median length was 74 feet (range: 25–88 feet).

Of the 30 injured workers whose age was known, the median age was 50 years (range: 23–73 years). Of the 20 injured workers with known work history, nine (45%) had ≥21 years of experience (Table 1). Of the 34 with known job positions, 15 (44%) were employed as masters (captains), and 19 (56%) as deckhands.

Eight injuries (23%) were fatal, although only two fatalities involved injuries that were not survivable (i.e., massive crushing injuries to the head and torso). No minor injuries were reported (Table 2). All 21 workers with moderate or serious injuries (4) survived; one of the six severely injured workers and five of the six critically injured workers died.

Injuries involving the deck winch drum (Figure) had a higher risk for fatal outcomes compared with injuries involving the winch cathead (RR = 7.5; 95% confidence interval [CI] = 1.1–53.7), but injuries involving the main winch (Figure) did not have an increased risk for fatal outcomes compared with the try-net winch (RR = 2.3; CI = 0.5–9.9).† Fatal outcomes also were associated with being alone on the vessel (RR = 5.8; CI = 2.1–15.9) and being alone on deck (RR = 4.0; CI = 1.2–13.6).

In 14 (41%) of the 35 cases, an item of loose clothing such as shorts, long sleeves, or gloves was cited as the first thing entangled in the winch. Nine of the 18 injuries to the upper extremities resulted in amputations; five of the upper extremity injuries were fractures. Six of the seven lower extremity injuries resulted in amputations; one amputation was fatal. Five of the seven workers with injuries to multiple body parts died (Table 2). Two workers who were alone on their vessels died from mechanical asphyxiation (i.e., compression of the chest by winch cables).

## Editorial Note

Deck winches are extremely hazardous mechanisms, with entanglements causing death to some workers and amputated limbs and other permanent disabilities to others. Among all U.S. fisheries, onboard injuries occurred most commonly in the Gulf of Mexico shrimp fishery (5). However, a search of the literature found only one previous study of injuries to workers in that fishery. The study found that, during 1986–2006, 19 patients with injuries involving shrimp winches were treated at a Texas hospital (6). All of the injuries were nonfatal, and 17 of the 19 affected the upper extremities. Injuries ranged in severity from crushed fingers to transhumeral amputations.

In August 2012, before the analysis described in this report was conducted, a deckhand aged 15 years on a commercial shrimp fishing vessel died in the Gulf of Mexico after becoming entangled in a winch (7); investigations of this fatality were ongoing as of March 8, and whether the youth had been working legally was unknown. The fatality of the young worker highlights the continuing workplace hazard found on many vessels employed in the U.S. fishing industry. In the 35 injuries described in this report, winch injuries occurred almost exclusively on side trawlers (vessels towing a trawl net from each side) and often involved experienced workers. Wearing loose fitting clothing was a contributing factor, as was working alone. Both masters (captains) and deckhands were vulnerable to winch injuries.

In 2005, a team of CDC injury epidemiologists and safety engineers collaborated to address the hazard of winch-related injuries on fishing vessels that use a purse seine (a large weighted net) in Alaska (8). An emergency stop button located strategically on the hydraulically powered winch was determined to be the most effective means for preventing winch injuries. The device was developed, tested, and licensed to a manufacturer for installation on new winches and for retrofitting on existing winches. A similar approach appears to be needed to develop viable prevention solutions to the unique hazards winches present on shrimp vessels.

The findings in this report are subject to at least two limitations. First, the absence of minor injuries and the small number of moderate injuries likely were a result of underreporting rather than actual characteristics of the distribution of injury severity in the population. Reporting to the U.S. Coast Guard of injuries requiring treatment beyond first aid is mandatory, but compliance with reporting less severe injuries is low. Data on these less severe injuries might have provided additional insights on the development of interventions. Second, large proportions of missing data on certain variables (such as race/ethnicity and work experience) might have introduced bias in calculations involving those variables.

Several interventions might help reduce or eliminate the shrimp winch entanglement hazard. An ideal solution is to remove the worker completely from the hazardous operation of manually guiding the cable. Installing and using hydraulic devices to guide the cable is an option, but the high cost might deter widespread adoption. Less ideal, but more affordable solutions might include minor modifications that could be made to existing deck machinery. Strong passive guarding could be attached around the main winch drums to prevent or reduce the severity of an entanglement injury. Welding extensions onto the cable guides would move the worker away from the cable.

Other interventions might involve mechanisms to stop the winch, either by disconnecting the power-take-off linkage, stopping the main engine, or disengaging the winch clutch arm. Further research is needed to develop and test the efficacy of these and other interventions. In addition to applying these safety interventions, crew members should be discouraged from working alone on deck, and training should include procedures for stopping the winch in an emergency and administering first aid (e.g., tourniquet use and cardiopulmonary resuscitation) for serious injuries such as those requiring amputations.

What is already known on this topic?

Fatalities in the fishing industry are most commonly the result of vessel disasters and falls overboard. Other fatal injuries, such as entanglement in fishing apparatus, are sustained onboard vessels while working on deck. The majority of onboard injuries in the U.S. fishing industry occur in the Gulf of Mexico.

What is added by this report?

During 2000–2011, a total of 35 work-related injuries, including eight fatal injuries, caused by deck winches occurred in the Southern shrimp fleet. Working alone and becoming entangled in the winch drum (as opposed to the winch cathead) were risk factors for fatal injury outcomes.

What are the implications for public health practice?

Further research is needed to develop effective and appropriate strategies for preventing winch injuries in the southern shrimp fleet. Potential interventions include improvements to cable winch guide systems, installing guards on the winch drums and catheads, and discouraging workers from working alone on deck.

## Figures and Tables

**FIGURE:**
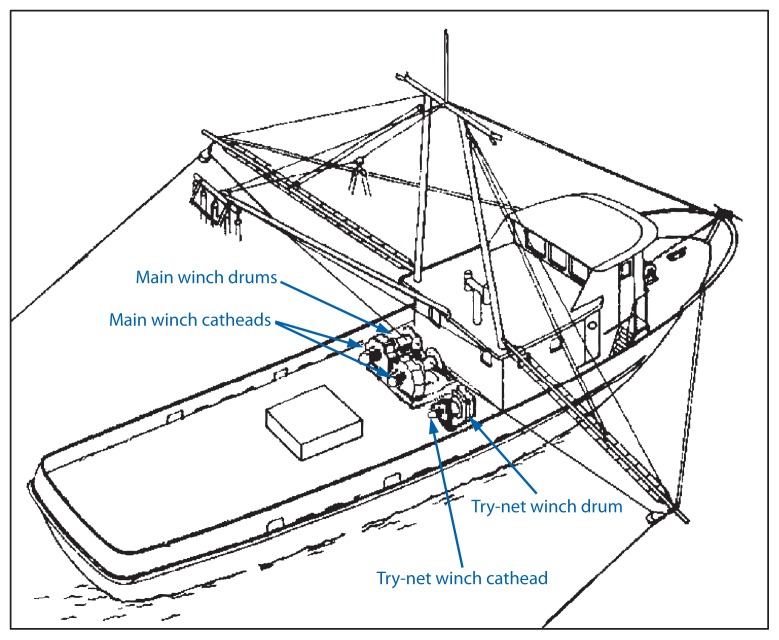
Typical deck layout, showing types of winches on a side trawler — Southern shrimp fleet, United States, 2000–2011

**TABLE 1:** Demographic characteristics of workers (N = 35) injured by deck winches — Southern shrimp fleet, United States, 2000–2011

Characteristic	No.	(%)*
**Age group (yrs)**		
<24	1	(3.3)
25–44	6	(20.0)
45–64	18	(60.0)
≥65	5	(16.7)
Unknown	5	—
**Sex**		
Male	34	(97.1)
Female	1	(2.9)
**Race/Ethnicity**		
White, non-Hispanic	7	(31.8)
Asian	11	(50.0)
Hispanic	4	(18.2)
Unknown	13	—
**Experience (yrs)**		
≤1	1	(5.0)
2–5	2	(10.0)
6–10	4	(20.0)
11–20	4	(20.0)
≥21	9	(45.0)
Unknown	15	—
**Job position**		
Master	15	(44.1)
Deckhand	19	(55.9)
Unknown	1	—

* Missing values were excluded from percentage distributions.

**TABLE 2:** Characteristics of injuries caused by deck winches, by fatal/nonfatal status — Southern shrimp fleet, United States, 2000–2011

Characteristic	Nonfatal (n = 27)		Fatal (n = 8)	
	
	No.	(%)*	No.	(%)
**Year of injury**				
2000	3	(11.1)	0	(0.0)
2001	5	(18.5)	0	(0.0)
2002	1	(3.7)	0	(0.0)
2003	2	(7.4)	2	(25.0)
2004	1	(3.7)	2	(25.0)
2005	1	(3.7)	1	(12.5)
2006	2	(7.4)	0	(0.0)
2007	0	(0.0)	0	(0.0)
2008	1	(3.7)	0	(0.0)
2009	4	(14.8)	0	(0.0)
2010	0	(0.0)	1	(12.5)
2011	7	(25.9)	2	(25.0)
**Injury severity**				
Minor	0	(0.0)	0	(0.0)
Moderate	5	(18.5)	0	(0.0)
Serious	16	(59.3)	0	(0.0)
Severe	5	(18.5)	1	(12.5)
Critical	1	(3.7)	5	(62.5)
Unsurvivable	—	—	2	(25.0)
**Body part**				
Head	1	(3.7)	0	(0.0)
Trunk	0	(0.0)	2	(25.0)
Upper extremities	18	(66.7)	0	(0.0)
Lower extremities	6	(22.2)	1	(12.5)
Multiple body parts	2	(7.4)	5	(62.5)
**Nature of injury**				
Amputations	14	(51.9)	1	(12.5)
Fractures	7	(25.9)	0	(0.0)
Compression asphyxia	0	(0.0)	2	(25.0)
Multiple unspecified	2	(7.4)	5	(62.5)
Other	4	(14.8)	0	(0.0)
**Source of injury**				
Winch drum	8	(34.8)	7	(87.5)
Winch cathead	15	(65.2)	1	(12.5)
Unknown	4	—	0	—

* Missing values were excluded from percentage distributions.

## Footnotes

* The Southern shrimp fleet covers a region of eight warm-water shrimp-producing states in the Gulf of Mexico and South Atlantic. The vessels are similar with respect to gear types, fishing processes, equipment, and hazards, and are represented by the Southern Shrimp Alliance, an organization that unites vessels across the many state and federal fishery management plans.

† Side-trawl shrimp vessels typically employ two main winches mounted in the same frame on the aft deck (Figure). These winches wind multiple strands of wire cable that tow the main trawl nets. A smaller third winch, the “try-net” winch, is similarly located and drags a small net used to sample the expected catch of the main trawls. The try-net winch is driven by an electric motor, whereas the two main winches are mechanically driven through a power-take-off linkage system from the vessel’s main engine. A cathead spool mounted on the end of each winch shaft spins when the power-take-off controlling lever is engaged. Each winch operator is required to manually guide the retrieved cable evenly onto the drum with a pivoting lever. Guiding the cable requires the operator to exert considerable force pushing and pulling the pivoting lever. Typically, few or no machine guards are present to prevent the worker from falling into or becoming entangled in moving machinery or the cable.
